# Elucidating the Molecular Interactions of Flavonoid–Metal Complexes via Isothermal Titration Calorimetry

**DOI:** 10.3390/biom16101526

**Published:** 2026-10-20

**Authors:** Martin Guerrero, Alexander Kim, Colby Braden, Yuping Bao

**Affiliations:** Department of Chemical and Biological Engineering, The University of Alabama, Tuscaloosa, AL 35487, USA; mguerrero1@crimson.ua.edu (M.G.); akim13@crimson.ua.edu (A.K.); cwbraden@crimson.ua.edu (C.B.)

**Keywords:** isothermal titration calorimetry, flavonoids, molecular interactions, flavonoid-metal complex

## Abstract

Flavonoids exhibit many proven health benefits and play critical roles in preventing, delaying, or even reversing chronic disease conditions. The biological activities of flavonoids strongly depend on their structures, and they interact with many biological components, such as proteins, nucleic acids, and inorganic ions. The coordination of metal–flavonoid complexes has previously been shown to increase the biological activity of flavonoids. Additionally, the complexation of metal ions and flavonoids serves as a great strategy to create advanced structures, such as nanoparticles or thin films. Among various metal ions, ferric ions are essential for many biological processes and have a high affinity for binding with flavonoids to form advanced structures. Therefore, understanding the molecular interactions between flavonoids and ferric ions (Fe(III)) is of great fundamental and practical importance. In this paper, using quercetin and its glycoside form, rutin, as model systems, we systematically investigated their complexation behaviors with Fe(III). The presence of a sugar molecule on rutin leads to differences between rutin and quercetin in solubility, stability, and the number of metallic ion coordination sites. Here, our studies specifically investigated the effects of solvents and temperature on their complexation behaviors with Fe(III) using isothermal titration calorimetry (ITC). The complexation processes of both flavonoids with Fe(III) were similar and primarily exothermic. However, the solvent significantly affected the thermodynamic parameters of the interactions. These findings offer valuable guidance for understanding the interactions between flavonoids and metal ions and provide insights into the development of flavonoid-based advanced structures.

## 1. Introduction

Flavonoids have many demonstrated health benefits [1,2,3,4,5,6], such as neuroprotection [7], anti-inflammatory effects [8,9], cardioprotection [10], anti-cancer properties [3,11,12], and insulin resistance prevention [13]. Some flavonoids also play significant roles in preventing, delaying, or reversing chronic disease conditions [14,15,16,17]. For example, dietary flavonoid intake is known to delay the biological aging process [17], slow down diabetes progression [13], and reverse drug resistance in cancer chemotherapy [18,19,20]. As naturally occurring phenolic compounds, flavonoids have very diverse structures, which are directly related to their biological activities [21,22]. The key structure of a flavonoid typically consists of two phenyl rings (A and B) and a heterocyclic ring (C) with different hydroxylation positions, glycosylation, and other substitutions [21,23]. This structural diversity leads to variation in their biological activities and specific interactions with biological systems. For example, quercetin is known to block P-glycoprotein (P-gp) pumps to reverse drug resistance [24,25,26,27], naringenin shows effective neuroprotection with the ability to penetrate the blood–brain barrier [28,29], and epigallocatechin-gallate inhibits glucose transporter member 5 (GLUT5) [30,31], a fructose-specific transporter that is often heavily utilized by cancer cells. Regardless of biological activities, flavonoids can interact with many biological components, including serum proteins, nucleic acids, and mineral ions [32,33]. Particularly, the chelation of flavonoids to various metal ions plays a critical role in managing oxidative stress, inflammation, and human diseases [6,34,35,36,37]. The metal–flavonoid complexation exhibited increased biological activities compared to free flavonoids [36,38,39,40], serving as potential drug candidates with better pharmacological activities [41]. Compared to other complexes, iron coordination is of special interest because of its essential role in many biological processes and relevance in various diseases. Flavonoids are known to modulate iron transport [42] and iron homeostasis [43,44]. Additionally, metal complexation also offers an effective strategy to create advanced structures [45,46,47]. Therefore, further understanding of the molecular interactions between flavonoids and metal ions is of great importance for both fundamental science and practical applications.

The formation of metal–flavonoid complexes is commonly studied using Fourier transform infrared spectrophotometry (FTIR) by monitoring M-O stretching vibration (442–486 cm^−1^) and the shift in the absorption band of UV-vis spectra [48]. Despite their effectiveness in examining complex formation, these two methods do not provide quantitative information on molecular interactions. In contrast, isothermal titration calorimetry (ITC) has been shown to be an effective tool for quantitatively studying the molecular interactions of flavonoids [49], allowing for the determination of binding affinity (*K*_d_), enthalpy change (Δ*H*), entropy change (Δ*S*), and stoichiometry (*n*). For example, ITC has been applied to study the interactions of flavonoids with serum albumin [50,51,52], amino acids [53,54], and nucleic acids [55]. However, very few ITC studies have been reported for the study of flavonoid-metal complexation [42,56].

Here, using quercetin and its glycoside form, rutin, as model systems, we systematically investigated the molecular interactions of quercetin/rutin with Fe(III) using ITC. The presence of a sugar molecule on rutin leads to differences between rutin and quercetin in solubility, stability, and the number of binding sites. Here, we specifically investigated the effects of solvents and temperature on their complexation behaviors with Fe(III). The complexation processes of quercetin/rutin with ferric ions were exothermic, with a similar trend. However, the solvent composition affected the binding process with distinct thermodynamic parameters. These findings offer valuable guidance for understanding the interactions between flavonoids and metal ions and provide insights into the development of flavonoid-based advanced structures.

## 2. Materials and Methods

### 2.1. Chemicals

Absolute ethanol (EtOH, 100%) was obtained from Thermo Fisher Scientific (Waltham, MA, USA); quercetin dihydrate (≥97%, Thermo Fisher Scientific, Ward Hill, MA, USA); rutin hydrate (≥97%, Thermo Scientific, Ward Hill, MA, USA); and iron (III) chloride anhydrous (FeCl_3_, 98%, Alfa Aesar, Haverhill, MA, USA). Dimethyl sulfoxide (DMSO, 99.9%, VWR Chemicals BDH, Radnor, PA, USA). All reagents were of analytical grade and used without further purification. Deionized (DI) water was used in all solution preparations.

### 2.2. ITC Study of Solvent Effect

The coordination of quercetin/rutin with ferric ion was studied using an affinity-based low-volume ITC instrument (TA Instruments Affinity ITC system, Lindon, UT, USA) to evaluate the effects of solvent composition and temperature. Stock solutions of quercetin and rutin were prepared separately by dissolving 33.83 mg of quercetin dihydrate (M.W. 338.3 g/mol) or 62.8 mg of rutin hydrate (M.W. 628.5 g/mol) in 10 mL of 100% ethanol (EtOH) to obtain a final concentration of 10 mM. The solutions were stirred for 2 days at 25 °C to ensure complete dispersion. The stock solutions were then diluted to a final flavonoid concentration of 1.5 mM using the appropriate volumes of absolute ethanol and DI water to reach final ethanol volume contents of 30%, 40%, 50%, 60%, 70%, or 75% (*v*/*v*) in the mixture. A ferric chloride (FeCl_3_) stock solution was prepared by dissolving 162.2 mg of anhydrous FeCl_3_ in 10 mL of deionized (DI) water. The FeCl_3_ stock was subsequently diluted to a final concentration of 15 mM using solvent mixtures with the same ethanol concentrations as those used for the corresponding flavonoid solutions. All flavonoid and FeCl_3_ solutions were allowed to equilibrate overnight before ITC measurements, and visible changes in solution appearance, including turbidity and color, were monitored as qualitative indicators of changes in solubility (Figures S1–S3). For each titration, 300 μL of the 1.5 mM quercetin or rutin solution was loaded into the ITC sample cell, which has an active cell volume of 182 μL. The injection syringe was filled with 15 mM FeCl_3_ prepared in the corresponding solvent mixture to ensure that the solvent composition was matched between the sample cell and titrant. All titrations were conducted at 25 °C with continuous stirring at 125 rpm. Each experiment consisted of 35 injections of 2.5 μL each. The pH values of all the solutions before and after titration were measured accordingly. Several control experiments were performed to assess potential background and dilution effects, including Fe(III) titration into the blank solvent, titration of the blank solvent into the flavonoid solution, reverse-order titration, and comparative dilution experiments using DMSO and ethanol.

### 2.3. ITC Study of Temperature Effects

To evaluate the effect of temperature on the binding interaction, the titration experiments conducted in 60% EtOH were repeated at 37 °C under otherwise identical experimental conditions. The temperatures of 25 °C and 37 °C were selected to characterize the interaction under both standard laboratory and physiological temperatures. Measurements at 25 °C provide a conventional reference for thermodynamic analysis and are consistent with the conditions used for the quercetin/rutin solubility studies. In contrast, measurements at 37 °C provide information on the interaction under physiological temperature and facilitate comparison with previous studies of flavonoid–iron interactions relevant to iron homeostasis and biological activity. Control experiments, including Fe(III) titration into the blank solvent and blank-solvent titration into the flavonoid solution, were also performed at 37 °C.

### 2.4. Data Analysis

Raw thermograms were processed and analyzed using the NanoAnalyze software (TA Instruments, Version 4.0.2.0). To account for the heat associated with FeCl_3_ dilution and Fe(III) hydrolysis in the solvent system, control experiments were performed by titrating the 15 mM FeCl_3_ solution into the corresponding blank solvent mixtures without flavonoid under otherwise identical experimental conditions for both 25 and 37 °C. The heats measured in these control experiments were subsequently subtracted from the corresponding raw reaction thermograms prior to peak integration and thermodynamic modeling. Baseline correction was applied to all datasets prior to curve fitting. Binding isotherms were generated by integrating the heat flow over time for each injection. Quercetin and rutin data were fit using multiple sites, but sequential two sites were also used to compare best fit using 99% confidence. Thermodynamic parameters, such as *K*_d_, Δ*H*, and Δ*S*, were extracted from the model fits.

### 2.5. UV-Visible Spectroscopy of the Complex

The complexation between flavonoids and ferric ions was further monitored using ultraviolet-visible (UV-Vis) absorption spectroscopy. A 20 μL aliquot of the reaction mixture was collected from the ITC sample cell and diluted with the same solvent mixture to a final volume of 2 mL. The solvent in the reference cell was changed accordingly to match the solvent composition of the sample cell. The resulting samples were analyzed in 1 cm path-length quartz cuvettes using a VWR V-3100PC UV-Vis spectrophotometer (Radnor, PA, USA) over a wavelength range of 200–900 nm. Changes in characteristic absorption features and spectral shifts were monitored to confirm flavonoid–ferric ion coordination formation.

## 3. Results and Discussion

Quercetin and rutin are both abundant flavonoids from plants with many reported health benefits [57,58,59], such as protective effects against cell oxidative stress [60] and ameliorative effects on fatty liver disease in mice [61]. The complexation with iron ions has shown additional benefits, such as enhanced DNA cleavage [36], reactive oxygen species scavenging [62], modulation of iron transport [42], and homeostasis [43,44]. Rutin and quercetin inhibited bacterial growth and biofilm formation primarily through iron chelation [63]. Despite similar reported biological effects, quercetin and rutin differ in chemical structure, with rutin having a bound sugar molecule at the C3 position (Figure 1). The highlighted sites in Figure 1 were selected based on the established O,O-donor coordination chemistry of flavonoids and previous experimental and theoretical studies of quercetin–metal interactions [64]. Quercetin contains three commonly proposed bidentate coordination regions: the 3′,4′-catechol group on the B ring, the 3-hydroxy-4-oxo group on the C ring, and the 5-hydroxy-4-oxo group spanning the A/C rings. The catechol moiety has been reported to be a particularly favorable coordination site for Fe(III) [65], although the relative contribution of the different sites depends on pH, solvent, metal-to-ligand ratio, and complex stoichiometry. In rutin, glycosylation at the C3 hydroxyl group prevents direct coordination through the 3-OH/4-oxo region, whereas the 3′,4′-catechol and 5-OH/4-oxo regions remain available for potential metal coordination. Thus, Figure 1 illustrates the principal potential coordination regions rather than assigning the individual binding transitions to specific molecular sites. Previous studies have suggested multiple protonation/deprotonation equilibria for quercetin with reported pKa values varying with experimental conditions [66]. However, the present ITC experiments were performed in ethanol–water mixtures and protonation constants were not independently determined under the experimental conditions, therefore, these literature pKa values only provide supporting chemical context for studying the coordination.

The highlighted locations also directly contribute to the biological activities of these molecules, with rutin being less active than quercetin [67]. For example, quercetin has demonstrated greater antibacterial activity [68,69] and a stronger chemosensitizing effect on doxorubicin treatment [70] than rutin. The observed differences in biological activity suggest that flavonoid glycosylation may also influence their ability to chelate iron. Quantitative evaluation of the interactions between quercetin, rutin, and iron ions could therefore advance our fundamental understanding of their biological activities while providing guidance for the development of advanced structures based on metal–flavonoid coordination [45]. In addition to influencing biological activity, glycosylation also alters the solubility and stability of flavonoids [71], properties that may directly affect their complexation with iron ions. Both quercetin and rutin exhibit limited solubility in water but are substantially more soluble in organic solvents, such as alcohols and DMSO [72]. Despite extensive studies of the biological activities of flavonoids and their metal complexes, relatively little quantitative information is available regarding the fundamental complexation processes between flavonoids and metal ions, particularly ferric ions. Therefore, this study focuses on the coordination of quercetin and rutin with iron ions using ITC. These two structurally related flavonoids provide an excellent model system for elucidating how glycosylation influences flavonoid–iron complexation. Furthermore, identifying the specific structural sites involved in metal coordination may provide valuable insights for the rational design of advanced structures based on metal–flavonoid interactions.

### 3.1. Solvent Effects

Because ITC directly measures the heat released or absorbed during an interaction, the thermal properties of the solvent and experimental temperature can substantially influence the measured heat signal. Here, the ITC experiments were intentionally performed without an added buffer because the objective of this study was to examine the overall calorimetric behavior of Fe(III)-quercetin and Fe(III)-rutin coordination in ethanol–water solvent systems. Introducing a conventional aqueous buffer into ethanol systems would introduce an additional chemical component and could itself interact with Fe(III) or alter Fe(III) hydrolysis, ligand protonation, and the solvent environment. Such effects would further complicate the interpretation of the solvent-dependent ITC measurements. In addition, the relatively high ethanol content required to maintain sufficient flavonoid solubility substantially limits the direct applicability of conventional aqueous buffering conditions. In this study, we investigated the effects of solvent composition and temperature on the complexation of quercetin and rutin with Fe(III). Understanding these effects provides insight into the fundamental interactions between flavonoids and iron ions and may facilitate the rational design of advanced metal–flavonoid structures and delivery systems. Ethanol and DMSO are among the most commonly used solvents for poorly water-soluble flavonoids. Our preliminary ITC experiments, however, showed substantially greater dilution and mixing heats for DMSO than for ethanol (Figure S4). A strong solvent background associated with DMSO has also been reported in ITC studies of ligand–protein interactions [73]. In this reported study, the addition of 10% (*v*/*v*) DMSO increased the calculated *K*_d_ from 5.588 to 13.02 μM and changed the free energy from −30.28 to −28.17 kJ mol^−1^. Therefore, ethanol–water mixtures were selected for subsequent experiments because ethanol enhances flavonoid solubility, while its relatively low specific heat capacity (2.46 J g^−1^ °C^−1^) is balanced by the higher specific heat capacity of water (4.184 J g^−1^ °C^−1^). At a given temperature, the solubility of quercetin and rutin generally increases with increasing ethanol content in ethanol–water mixtures [74,75]. Previous studies have also demonstrated that solvent composition can directly influence the interaction of quercetin with bovine serum albumin [76], and the formation of quercetin-hydroxypropyl-β-cyclodextrin host–guest complexes [77]. These findings suggest that solvent composition may similarly influence the coordination of flavonoids with Fe(III) ions. To ensure sufficient flavonoid solubility while systematically evaluating solvent effects, the ethanol volume fraction varied from 30% to 75% (*v*/*v*). The selected solvent compositions were intended to represent three regimes: low ethanol content (30% and 40%), in which flavonoid solubility may be limited; intermediate ethanol content (50% and 60%), which provides sufficient flavonoid solubility while maintaining a balanced solvent composition; and high ethanol content (70% and 75%), in which the thermal properties of the system are increasingly dominated by ethanol. After establishing the solvent composition, ITC measurements could, in principle, be performed by titrating either the flavonoid solution into the Fe(III) solution or Fe(III) solution into the flavonoid solution. Although our previous synthesis of quercetin–Fe complex nanoparticles involved adding quercetin to an Fe(III) solution, reproducible ITC thermograms could not be obtained when flavonoid solutions were titrated into Fe(III) solutions (Figure S5). Therefore, all subsequent ITC experiments were performed by titrating Fe(III) into flavonoid solutions. Figure 2 shows the thermograms and isotherms of ITC experiments by titrating 2.5 μL per injection of Fe(III) solution (15 mM) into 1.5 mM quercetin in ethanol–water solution with various ethanol percentages. Here, Fe(III) was dissolved in the same solvent as for flavonoid solution and allowed to equilibrate overnight before ITC experiments.

The ITC thermograms represent the raw calorimetric data and record the rate of heat released or absorbed following each injection. The measured heat signal reflects multiple processes, including flavonoid and Fe(III) dilution and mixing, Fe(III) hydrolysis, solvent reorganization, and flavonoid–Fe(III) coordination. Therefore, the thermograms provide information about the overall heat associated with the injection and reaction processes rather than exclusively representing the coordination reaction. The height and area of each peak reflect the magnitude and total amount of heat, respectively, associated with the processes occurring after each injection. As the system approaches equilibrium or saturation, the peak magnitude generally decreases because fewer additional coordination events occur following subsequent injections. Importantly, larger peaks indicate a greater heat change but do not necessarily correspond directly to a higher binding affinity, as the observed signal is also influenced by the binding enthalpy and other heat-producing or heat-absorbing processes. The ITC thermograms clearly demonstrate a strong effect of solvent composition on the measured calorimetric response. At 30% ethanol, the heat-rate peaks reached approximately 90 μJ/s, substantially higher than those observed at the other ethanol concentrations. This large signal likely reflects a significant contribution from flavonoid dilution and mixing because quercetin may not have been completely dissolved at the lower ethanol content. Under these conditions, the calorimetric signal may therefore be dominated by dilution and solvent-mixing processes rather than representing a discrete coordination transition. As the ethanol content increased, the peak intensity decreased substantially to approximately 15 μJ/s. At intermediate ethanol concentrations (50% and 60%), the thermograms exhibited well-defined binding isotherms and clear progression toward equilibrium, providing the most suitable conditions for quantitative analysis of the quercetin–Fe(III) interaction. At higher ethanol concentrations (≥70%), the peak areas decreased further. This behavior may result from changes in solvent properties, including solvent heat capacity, Fe(III) solvation and hydrolysis, and the thermodynamics of flavonoid–Fe(III) coordination. Because the Fe(III) concentration and injection volume were held constant across all experiments, the progressive decrease in peak area indicates a reduction in the net heat associated with each Fe(III) injection as the solvent became increasingly ethanol-rich. This observation is consistent with a reduced overall exothermic driving force for complex formation. However, the observed decrease may arise from several factors, including reduced binding enthalpy, changes in binding affinity, diminished Fe(III) hydrolysis, or competing contributions from Fe(III) desolvation and solvent reorganization. Therefore, the thermograms alone cannot distinguish among these mechanisms. Because the same number of injections were used in each experiment, differences in the total duration of the calorimetric response provide additional qualitative information about the progression toward equilibrium. For example, the experiment conducted in 60% ethanol exhibited the longest overall response, extending to approximately 5000 s, whereas the signal at 70% ethanol returned to the baseline earlier, at approximately 3500 s, compared with approximately 4300–5000 s for the other solvent compositions. This earlier return to baseline suggests that the system reached equilibrium or saturation with fewer effective injections under the high-ethanol condition. Possible explanations include changes in the solubility or stability of the Fe(III)-quercetin complexes and altered Fe(III) speciation in the lower-water environment. Further quantitative analysis of the integrated heats and fitted binding parameters is required to distinguish between these possibilities.

The raw ITC thermograms provide valuable information about the thermodynamics and potential mechanisms underlying the coordination reaction. Typically, large initial peaks indicate a substantial heat change associated with the interaction, while a rapid decrease in peak magnitude may indicate progression toward saturation. The observed exothermic peaks are consistent with the formation of Fe-O coordination bonds and may also involve the deprotonation of phenolic hydroxyl groups during complex formation. Based on the molecular structures of quercetin and rutin and previous studies of flavonoid–Fe(III) interactions, the catechol moiety has frequently been reported as a favorable Fe(III)-coordination site, although the preferred coordination mode can depend on solution conditions and complex stoichiometry [65,78]. In contrast, the 5-OH/4-oxo site may exhibit moderate reactivity. The observed calorimetric response may therefore reflect the formation of one or more inner-sphere coordination complexes involving these functional groups. A well-defined sigmoidal binding transition is generally required to reliably extract thermodynamic parameters using a single-site or other simple binding model. Among the solvent conditions investigated, the ITC data obtained in 60% ethanol exhibited the most clearly defined transition and were therefore considered the most suitable for quantitative fitting. Consequently, the thermodynamic parameters derived from the experiments conducted in 60% ethanol were used for the subsequent analysis and discussion.

Figure 3 shows the ITC thermograms for the titration of Fe(III) into rutin solutions under the same six ethanol–water conditions used for quercetin. Although rutin and quercetin share a similar flavonoid backbone, the sugar moiety present in rutin is expected to influence its solubility and coordination behavior. Unlike quercetin, rutin did not exhibit a substantially elevated heat-rate peak at 30% ethanol. Instead, the peak amplitude was approximately 22 μJ/s at 30% ethanol and decreased to a broad minimum of approximately 13–14 μJ/s at the other ethanol concentrations. This difference is likely attributable to the greater solubility of rutin, which reduces the contribution of dilution and mixing effects to the overall calorimetric signal. The catechol group on the B-ring is considered a primary coordination site for Fe(III) in both quercetin and rutin. In quercetin, the adjacent C3-OH and C4-oxo groups provide an additional potential high-affinity coordination site. In rutin, however, the C3 hydroxyl group is glycosylated, effectively limiting the availability of this site for metal coordination. The C5-OH/C4-oxo group remains available in both molecules but is generally considered to exhibit lower coordination affinity than the catechol group. These structural differences provide a basis for comparing how glycosylation influences the coordination behavior of quercetin and rutin with Fe(III).

A notable feature of the Fe(III)-rutin thermograms is the progressive shift in the calorimetric response toward less exothermic and, under some conditions, net endothermic behavior as additional Fe^3+^ was injected. This behavior represents more than a simple difference in signal magnitude and suggests that the relative contributions of the underlying processes change with solvent composition. Because the 3-OH position in rutin is glycosylated, Fe(III) coordination is expected to occur primarily through the 3′,4′-catechol group and the 5-hydroxy-4-keto site. Compared with quercetin, the loss of the 3-OH/4-oxo coordination site may therefore alter both the coordination chemistry and the overall enthalpic response of the system. At 30% ethanol, the initial heat-rate peaks were relatively large and progressively decreased with successive injections. The integrated isotherm reached a plateau at approximately 15 kJ mol^−1^. The integrated isotherm progressively increased, reaching a plateau at approximately 0 kJ mol^−1^, indicating a substantial net endothermic calorimetric response. At 40% ethanol, the overall response was also endothermic but smaller, reaching a plateau of approximately −2 kJ mol^−1^. In contrast, the experiment conducted at 50% ethanol produced an approximately linear increase in integrated heat from about −12 to 1 kJ mol^−1^ without a well-defined smooth saturation transition. This behavior suggests that the measured heat at 50% ethanol may contain substantial contributions from processes other than a simple Fe(III)-flavonoid binding event and should therefore be interpreted cautiously. A substantially different response was observed at higher ethanol concentrations. At 60% ethanol, the integrated isotherm exhibited a well-defined sigmoidal transition, changing from approximately −8 to 0 kJ mol^−1^ before reaching a plateau at higher molar ratios. The 70% ethanol condition showed a similar but less pronounced transition, ranging from approximately −8 to −0.5 kJ mol^−1^. In contrast, the response at 75% ethanol remained predominantly exothermic, increasing from approximately −10 to −2 kJ mol^−1^. Together, these results demonstrate that increasing the ethanol content changes not only the magnitude but also the sign and shape of the net calorimetric response.

These solvent-dependent changes are consistent with substantial differences in the relative contributions of ligand dilution, Fe(III) hydrolysis and speciation, desolvation, protonation, and Fe(III)-rutin coordination. The behavior of rutin differs markedly from that observed for quercetin under comparable conditions. Quercetin exhibited predominantly exothermic responses between 50% and 75% ethanol, with more clearly defined binding transitions at intermediate-to-high ethanol concentrations. The more consistently exothermic response of quercetin may reflect its additional metal-binding functionality, particularly the availability of the 3-OH/4-oxo coordination site. In contrast, the calorimetric response of rutin shows a stronger dependence on solvent composition, suggesting that changes in Fe(III) speciation, ligand solvation, protonation, and desolvation make substantial contributions to the measured heat. These results demonstrate that quercetin and rutin exhibit distinct thermodynamic responses during their interactions with Fe^3+^. However, differences in peak magnitude or the sign of the observed heat alone should not be interpreted as direct evidence of differences in binding affinity. Quantitative comparisons should instead be based on binding constants, enthalpy, entropy, and stoichiometry derived from appropriate ITC models and supported by suitable background controls (Figures S6–S9).

The Fe(III)-rutin thermograms exhibited a stronger dependence on solvent composition than those obtained for quercetin. Under some conditions, the calorimetric response shifted toward less exothermic or net endothermic behavior, reflecting changes in the overall balance of Fe(III) hydration and desolvation, ligand solvation, proton transfer, and coordination. The appearance of endothermic contributions may therefore be associated with processes such as disruption of the Fe(III) hydration shell and conformational or structural reorganization during complex formation. A plateau in the integrated heat suggests that the system approached an apparent saturation or equilibrium state under the experimental conditions, whereas the absence of a well-defined plateau may indicate that multiple processes, including continued complex or network formation, contribute to the measured heat. Compared with quercetin, rutin generally exhibited smaller and more solvent-dependent calorimetric responses. These differences are consistent with the structural consequences of glycosylation at the 3-position. Quercetin contains three potential Fe(III)-coordination regions: the 3′,4′-catechol group on the B ring, the 3-hydroxy-4-keto site on the C ring, and the 5-hydroxy-4-keto site. In rutin, glycosylation blocks the 3-hydroxyl group, leaving the catechol and 5-hydroxy-4-keto sites as the primary potential coordination regions. Thus, rutin retains substantial Fe(III)-chelating capability, but glycosylation alters the available coordination environment and the associated thermodynamic response.

Overall, the ITC data suggests that glycosylation does not eliminate Fe(III) coordination but modifies its calorimetric signature and solvent dependence. The apparent Fe stoichiometry remained broadly similar, approximately 1:3–4, although the transitions were less clearly resolved under some solvent conditions. These results are consistent with rutin retaining Fe(III) coordination through its catechol and 5-hydroxy-4-keto sites while lacking the contribution associated with the 3-hydroxy-4-keto site in quercetin. Previous studies have likewise shown that glycosylation can influence flavonoid–iron coordination; for example, 7-*O*-glycosylation was reported to enhance iron coordination at the 4–5 site, whereas additional 6″-*O*-acylation did not produce a similar effect [79].

Solvent composition is also expected to influence flavonoid solubility, aggregation, and coordination behavior. Ethanol–water mixtures provide an intermediate solvent environment that can support the dissolution of both relatively hydrophobic aglycones and more polar glycosylated flavonoids. Previous studies have shown that the ethanol/water ratio affects the extraction and antioxidant properties of phenolic compounds [80]. In addition, poorly soluble quercetin may form aggregates or crystals under supersaturated conditions [81]. These solvent-dependent effects may contribute to the differences observed in the ITC thermograms and should therefore be considered when interpreting the apparent thermodynamic parameters.

### 3.2. Temperature Effect

Besides solvent composition, temperature can also influence ITC measurements for a specific binding system [82], because temperature directly affects the equilibrium constant and, consequently, the apparent *K_d_*, Δ*H*, Δ*S*, and other thermodynamic parameters. Changes in temperature can also alter the relative contributions of binding, desolvation, proton transfer, and other coupled processes to the measured heat signal. Therefore, temperature-dependent ITC experiments can provide additional insight into the thermodynamics and mechanisms underlying molecular interactions [83]. For this study, temperature was also expected to impact the flavonoid solubility and, consequently, the availability of flavonoid molecules for coordination. The solubility of quercetin generally increases with increasing temperature and with increasing alcohol content in water–alcohol mixtures [74,75]. Although many ITC studies of biologically relevant interactions are performed at physiological temperature, measurements at 25 °C provide a useful reference condition and are directly relevant to the room-temperature formation of flavonoid–metal structures. Previous studies of quercetin, rutin, and other polyphenol interactions with iron have primarily focused on different solvent systems and experimental conditions, limiting direct comparison among reported results.

To evaluate the effect of temperature on flavonoid–Fe(III) interactions, ITC experiments were also performed at 37 °C under identical conditions in 60% ethanol–water (Figure 4). At 25 °C, both quercetin and rutin exhibited well-defined exothermic responses with discernible transitions in the integrated thermograms. Increasing the temperature to 37 °C substantially reduced the heat signals and produced less well-resolved isotherms, particularly for rutin, for which the integrated response became nearly linear. Thus, a discrete binding transition could not be reliably resolved for rutin at 37 °C under these conditions. These temperature-dependent changes may reflect differences in Fe(III) speciation, ligand solvation, and coordination equilibria. Because the 37 °C responses were less well resolved, the reduced calorimetric signals should not be interpreted as evidence of altered binding affinity alone.

The different responses of quercetin and rutin may also reflect their structural differences. In particular, glycosylation of the 3-OH group in rutin alters the availability of a potential Fe(III)-coordination site and may influence ligand solvation and conformational behavior during complex formation [84]. However, differences in peak magnitude or apparent enthalpy alone do not provide direct evidence for differences in binding affinity.

Collectively, the ITC results demonstrate a pronounced temperature dependence in the calorimetric responses of both quercetin and rutin. At 25 °C, both flavonoids exhibited well-defined exothermic transitions that were suitable for quantitative thermodynamic analysis. Increasing the temperature to 37 °C substantially reduced the heat signals and resulted in less well-resolved isotherms, particularly for rutin. These changes may reflect temperature-dependent effects on Fe(III) speciation, ligand and metal-ion solvation, and coordination equilibria. Because the 37 °C isotherms were less well resolved, changes in the apparent binding parameters should be interpreted cautiously and should not be attributed to changes in binding affinity alone.

The different responses of quercetin and rutin are consistent with the influence of molecular structure on their Fe(III) interaction behavior. In particular, glycosylation of the C3-OH group in rutin alters the availability of a potential coordination site and may also affect ligand solvation and conformational behavior. Thus, both molecular structure and experimental conditions, including temperature and solvent composition, should be considered when interpreting flavonoid–Fe(III) interactions. The present results provide a basis for further investigation of these complexes under more physiologically relevant solution conditions using complementary approaches to independently characterize their formation equilibria and coordination structures.

Beyond the raw thermograms, ITC provides quantitative parameters for flavonoid–Fe(III) interactions, such as *K*_d_, Δ*H*, Δ*S*, and apparent stoichiometry (*n*). The reliability of these parameters depends strongly on the appropriateness of the selected fitting model [85]. Because both quercetin and rutin contain multiple potential Fe(III) coordination sites, a sequential binding model was initially evaluated. However, this model produced binding constants that differed by several orders of magnitude, suggesting that it did not adequately describe the experimental data. A study of quercetin–Fe(III) complexation using NMR and computational analysis under controlled aqueous conditions did not identify a clear exclusive preference for a single coordination site [86], supporting the chemical plausibility of multiple-site binding. Therefore, a multiple-site model was subsequently used to analyze the ITC data, as shown in Figure 2, Figure 3 and Figure 4. Previous studies have generally shown that quercetin exhibits stronger iron-chelating capability than rutin, although the preferred coordination sites depend on the metal ion, solvent environment, and complex stoichiometry [63,65]. The 4-keto/5-hydroxy and 3-hydroxy/4-keto regions can participate in metal coordination, whereas catechol groups are considered particularly favorable sites for Fe(III) binding [65,78,87]. These structural considerations support the interpretation that the distinct thermodynamic behavior of quercetin and rutin arises, at least in part, from differences in the availability of their coordination sites.

The signs and magnitudes of Δ*H* and Δ*S* can provide additional insight into the forces contributing to the overall interaction. Previous studies have associated positive Δ*H* and Δ*S* values with hydrophobic contributions, negative Δ*H* and Δ*S* values with van der Waals and hydrogen-bonding interactions, and negative Δ*H* with positive Δ*S* values with electrostatic contributions [88]. However, because Fe(III)-flavonoid complexation may involve simultaneous coordination, proton transfer, desolvation, and solvent reorganization, these thermodynamic signatures should be interpreted as reflecting the overall interaction rather than a single, isolated binding force.

Table 1 summarizes the apparent thermodynamic parameters obtained by fitting the ITC data to the multiple-site fitting model for the interactions of quercetin and rutin with Fe(III) in 60% ethanol–water at 25 °C and 37 °C. Here, only a 60% ethanol–water solvent mixture was used to derive the thermodynamic parameters, because other conditions did not generate a meaningful fit (Table S1). The Fe(III)-flavonoid system likely involves several overlapping coordination at more than one chelating motif; therefore, the fitted parameters only represent apparent descriptors of the calorimetric response but may not be assigned to a specific molecular coordination site. For example, it was shown that quercetin–Fe(III) complexation in aqueous solutions involved multiple coordination sites, and the number of accessible sites depended on solution conditions such as pH [89]. Because of the overlapping coordination, here we used two interaction components to represent the two fitted sites. Δ*S* was derived from Δ*G* = RT ln *K*_d_ and Δ*H*, so it carries at least the uncertainty of Δ*H*.

For quercetin, at 25 °C, the two fitted components could not be distinguished by apparent affinity (*K*_d1_ ≈ *K*_d2_ ≈ 1.0 mM). The reported uncertainty in *K*_d1_ (±1 × 10^−9^ M) is unrealistically small given the large uncertainties in *n*_1_ (0.1 ± 1) and Δ*H*_1_ (+100 ± 200 kJ mol^−1^). It most likely means that *K*_d1_ reached a fitting boundary or was effectively fixed, not that it was precisely determined. The first component is therefore essentially unresolved, and its large positive Δ*S*_1_ (309 J mol^−1^ K^−1^) cannot be interpreted with confidence. The second component accounted for most of the apparent stoichiometry (*n*_2_ = 3 ± 1) and was modestly exothermic (Δ*H*_2_ = −20 ± 40 kJ mol^−1^), with a near-zero entropy term (−2 J mol^−1^ K^−1^). However, Δ*H*_2_ is itself only loosely constrained. Together, the identical *K*_d_ values and the poorly defined first component indicate that the 25 °C quercetin isotherm contains limited information to support a two-component description. At 37 °C, the two quercetin components became distinguishable. The first component had *K*_d1_ = (1.0 ± 0.4) × 10^−3^ M, *n*_1_ = 10 ± 2, Δ*H*_1_ = −5.0 ± 0.6 kJ mol^−1^, and Δ*S*_1_ = +41 J mol^−1^ K^−1^. This is the best-constrained quercetin component and describes a weakly exothermic, entropy-assisted process. An apparent stoichiometry of about 10 is much larger than a discrete coordination number. It more plausibly reflects processes such as the formation of polynuclear or aggregated Fe(III) quercetin species or deviations in the effective concentrations of the reacting species. In fact, our previous study has shown the quercetin–Fe complex nanoparticle formation in an ethanol–water mixture [47]. The second component showed a higher apparent affinity, with *K*_d2_ = (8 ± 6) × 10^−5^ M (≈0.08 mM). Its Δ*H*_2_ (−60 ± 40 kJ mol^−1^) was more exothermic than at 25 °C and was largely offset by an unfavorable entropy term (Δ*S*_2_ = −125 J mol^−1^ K^−1^), a pattern consistent with enthalpy–entropy compensation. Notably, this apparent affinity increased with temperature even though the process was exothermic. That is opposite to what the van’t Hoff relation predicts for a single equilibrium with a temperature-independent Δ*H*. The fitted component therefore probably does not represent the same process at both temperatures.

For rutin, at 25 °C, the first rutin component had *K*_d1_ = (2.1 ± 0.6) × 10^−4^ M, *n*_1_ = 2.6 ± 0.9, Δ*H*_1_ = −10 ± 1 kJ mol^−1^, and Δ*S*_1_ = +36 J mol^−1^ K^−1^. This is the best-defined parameter set in Table 1. It describes a modestly exothermic interaction (Δ*G* ≈ −21 kJ mol^−1^) in which the enthalpic (Δ*H* ≈ −10 kJ mol^−1^) and entropic (−TΔ*S* ≈ −11 kJ mol^−1^) terms contribute about equally. The second component was poorly resolved: *K*_d2_ = (1.0 ± 0.4) × 10^−3^ M, *n*_2_ = 1 ± 2, Δ*H*_2_ = +10 ± 100 kJ mol^−1^, and Δ*S*_2_ = +80 J mol^−1^ K^−1^. At 37 °C, the apparent *K*_d1_ of rutin increased to (1.0 ± 0.7) × 10^−3^ M, about a fivefold weaker apparent affinity, with *n*_1_ = 1.7 ± 1.0. The direction of this change is consistent with an exothermic interaction. However, Δ*H*_1_ was no longer constrained (−10 ± 200 kJ mol^−1^), so the associated Δ*S*_1_ (27 J mol^−1^ K^−1^) cannot be evaluated reliably. The second component returned an apparent *K*_d2_ of (2 ± 9) × 10^−8^ M. Because this uncertainty exceeds the fitted value, the result does not constitute evidence for a nanomolar-affinity interaction. The accompanying *n*_2_ (0.4 ± 1.1), Δ*H*_2_ (0 ± 300 kJ mol^−1^), and Δ*S*_2_ (122 J mol^−1^ K^−1^) are likewise unresolved. These large uncertainties point to strong parameter correlation, and they indicate that the 37 °C rutin data do not support two independently resolvable components.

Despite these uncertainties, several differences between the two flavonoids were clear. At 25 °C, the well-defined first component of rutin showed a higher apparent affinity (0.21 mM) than either quercetin component (≈1.0 mM). The two compounds also responded to temperature in different ways. The best-defined rutin component weakened from 25 to 37 °C. In contrast, the second quercetin component strengthened and became more exothermic, and quercetin showed a large apparent stoichiometry at 37 °C. The fitted *n* values should not be read as coordination numbers because they depend on the model and are sensitive to overlapping binding events and to the shape and resolution of the isotherms. The structural difference between the two flavonoids offers a plausible basis for their distinct behavior. In rutin, the rutinose substituent at C3 blocks the 3-hydroxy/4-carbonyl chelating motif. The glycoside also alters the ligand’s solvation, conformational flexibility, and the accessibility of the remaining 5-hydroxy/4-carbonyl and 3′,4′-catechol donor sites. Quercetin retains all three motifs, which could favor multiple coordination modes and Fe(III)-bridged oligomeric species. That possibility is consistent with, though not demonstrated by, its large apparent stoichiometry at 37 °C. The present ITC data alone cannot assign either fitted component to a specific coordination site, as is also seen in the replicates of the ITC (Figures S10 and S11).

Overall, raising the temperature from 25 to 37 °C altered the apparent thermodynamic profiles of both flavonoids, but the direction and magnitude of the changes differed between quercetin and rutin. The large uncertainties in several *K*_d_, *n*, Δ*H*, and Δ*S* values limit the mechanistic weight that individual parameters can offer. The data therefore show that temperature modulates the apparent calorimetric behavior of Fe(III)-flavonoid interactions, but they do not by themselves establish a specific molecular mechanism or a definitive change in intrinsic binding affinity. Complementary measurements would help resolve protonation equilibria, Fe(III) speciation, coordination stoichiometry, and the molecular origin of the temperature dependence. Subsequently, UV-vis spectra were collected for all the resulting reaction mixtures.

### 3.3. UV-vis Analysis of Flavonoid–Fe(III) Complexes

UV-vis has been widely used to study flavonoid–metal complexation, where the peak position provides valuable information on the involvement of the binding site in complexation [45]. Therefore, the UV-vis spectra of the reaction products obtained from ITC titration experiments (Figure 2, Figure 3 and Figure 4) were measured to further evaluate the effects of solvent composition and temperature on quercetin–Fe(III) and rutin–Fe(III) interactions (Figure 5).

The UV-vis spectrum of quercetin in 60% ethanol–water exhibits two characteristic absorption bands, centered near 255 and 370 nm [38], corresponding broadly to its benzene-ring and conjugated cinnamoyl-type electronic transitions, respectively. After titration of Fe(III) into flavonoid solutions, the spectra of the reaction products after showed considerable changes in both the intensities and shapes of these bands (Figure 5A). A continuous attenuation of the 370 nm band was observed with increasing ethanol content in the solvent mixtures. Because the 370 nm transition is sensitive to the electronic structure of the quercetin conjugated system, these changes indicate that the local electronic environment of quercetin is strongly influenced by solvent composition and Fe(III) coordination. At all solvent compositions, a new, prominent absorption peak at 293 nm was observed, which was the main peak for quercetin–Fe complex nanoparticles [47,86]. Additionally, a broad tailing absorption region extending past 400 nm up to approximately 500 nm appeared, a typical band representing ligand-to-metal charge transfer (LMCT) or ***π*** → π* transition resulting from chelation at the catechol (3′,4′-OH) or 3-hydroxy-4-keto sites [90,91]. The overall spectral profile of the quercetin–Fe(III) products was largely preserved for all ethanol compositions (Figure 5A), suggesting that similar reaction products were formed over this solvent range. However, differences in the spectral intensity, especially the 400–500 nm broad absorption region, indicated that solvent composition influenced reaction products. Increasing the reaction temperature from 25 to 37 °C did not substantially alter absorption features of the quercetin–Fe(III) products (Figure 5C), suggesting the overall electronic environment of the dominant reaction product was similar, but the relative distribution or stability of species in solution may vary. Because of the various pK values of a single quercetin molecule, the pH may affect the complexation and subsequent UV-vis spectra; thus, we have measured the pH of all resulting solutions (Table S2), where all the titration product solutions had similar pH around 2.5 at 25 °C.

In contrast, the rutin–Fe(III) reaction products in all solvent compositions at 25 °C showed similar absorption to that of the free rutin (Figure 5B). Free rutin exhibits characteristic absorption peaks at 265 nm and 354 nm [92,93]. After interaction with Fe(III), only slight shifts in the absorption peaks were observed (265 → 262 nm and 354 → 360 nm). This observation was different from reported large shifts in rutin–metal complexes [90,94]. However, the magnitude of this shift is highly pH-dependent; highly acidic environments are known to result in much smaller spectral shifts for rutin–metal complexes [95], which perfectly aligns with the low pH (~2.5) of our titration solutions. Compared to the quercetin–Fe(III) complex, the peak at 293 nm was at much lower intensity, and the broad visible-region absorption was barely seen. In rutin, the 3-OH group is substituted with a bulky rutinoside moiety, which prevents direct coordination through the 3-hydroxy-4-keto site that is available in quercetin. However, the products in 60% ethanol–water at 37 °C showed very distinct absorption peaks C (Figure 5D).

Overall, the UV-vis results complement the ITC measurements by demonstrating that both solvent composition and temperature influence the electronic environment and solution-state properties of the quercetin–Fe(III) and rutin–Fe(III) reaction products. The distinct spectral responses of quercetin and rutin further support the conclusion that glycosylation influenced their interactions with Fe(III).

## 4. Conclusions

This study demonstrates that subtle structural differences between flavonoids can substantially influence their interactions with Fe^3+^ and that these interactions are strongly dependent on the solvent and temperature conditions under which they are evaluated. By combining ITC with UV-vis spectroscopy, we characterized the thermodynamic and solution-state properties of quercetin–Fe(III) and rutin–Fe(III) reaction products across a range of ethanol–water compositions and temperatures. Among the solvent conditions examined, 60% ethanol provided the most well-defined binding isotherms, offering a suitable balance between flavonoid solubility and a measurable calorimetric response.

Quercetin and rutin exhibited distinct thermodynamic and spectroscopic behaviors that reflect the influence of C3 glycosylation on their Fe^3+^ coordination. The free 3-OH group in quercetin provides an additional potential coordination site that is unavailable in rutin because of the rutinoside substitution at the C3 position. Consequently, quercetin displayed a more clearly differentiated two-site thermodynamic profile, whereas rutin exhibited greater enthalpy–entropy compensation and a stronger dependence of its calorimetric response on experimental conditions. The distinct UV-vis spectral features of the two systems further support differences in their electronic and coordination environments, although the specific coordination sites cannot be assigned definitively from the present data alone. Temperature also substantially influenced the apparent thermodynamics of both flavonoid–Fe(III) systems. Increasing the temperature from 25 to 37 °C enhanced the apparent binding affinities obtained from the fitted ITC models, but the thermodynamic responses of quercetin and rutin differed markedly. Quercetin retained a relatively similar overall binding profile, with temperature altering the relative enthalpic and entropic contributions of the fitted interactions. In contrast, rutin showed a more pronounced reorganization of its thermodynamic profile, highlighting the potential importance of glycosylation, solvation, and temperature-dependent changes in Fe(III) coordination chemistry. These results emphasize that thermodynamic parameters measured under standard laboratory conditions may not fully predict the behavior of flavonoid–metal interactions under physiological temperature conditions.

More broadly, this work establishes a framework for using ITC to examine how molecular structure and environmental conditions jointly regulate metal–flavonoid interactions. Future studies integrating ITC with complementary structural and analytical techniques, such as mass spectrometry, spectroscopy, potentiometric studies, and structural characterization of the resulting complexes, will be important for identifying the specific coordination modes and distinguishing discrete molecular complexes from higher-order assemblies. For example, future studies combining ITC with potentiometric measurements will be valuable for independently determining the metal-ligand formation equilibria of the flavonoid–Fe(III) complexes. Such complementary measurements could provide formation/stability constants under defined solution conditions and further clarify the relationship between ligand protonation, coordination behavior, and the binding thermodynamics observed by ITC. Extending this approach to additional flavonoids, metal ions, and biologically relevant solution conditions may also reveal structure–thermodynamic relationships that can guide the rational design of metal–flavonoid complexes and nanostructures.

Ultimately, understanding how glycosylation, solvent composition, and temperature govern Fe(III) coordination provides a foundation for the development of flavonoid-based systems for iron chelation, antioxidant applications, metal delivery, and advanced metal–polyphenol nanostructures. These findings further highlight the importance of evaluating both the intrinsic molecular structure and the environmental context when translating flavonoid–metal interactions from fundamental thermodynamic studies to biological and materials applications.

## Figures and Tables

**Figure 1:**
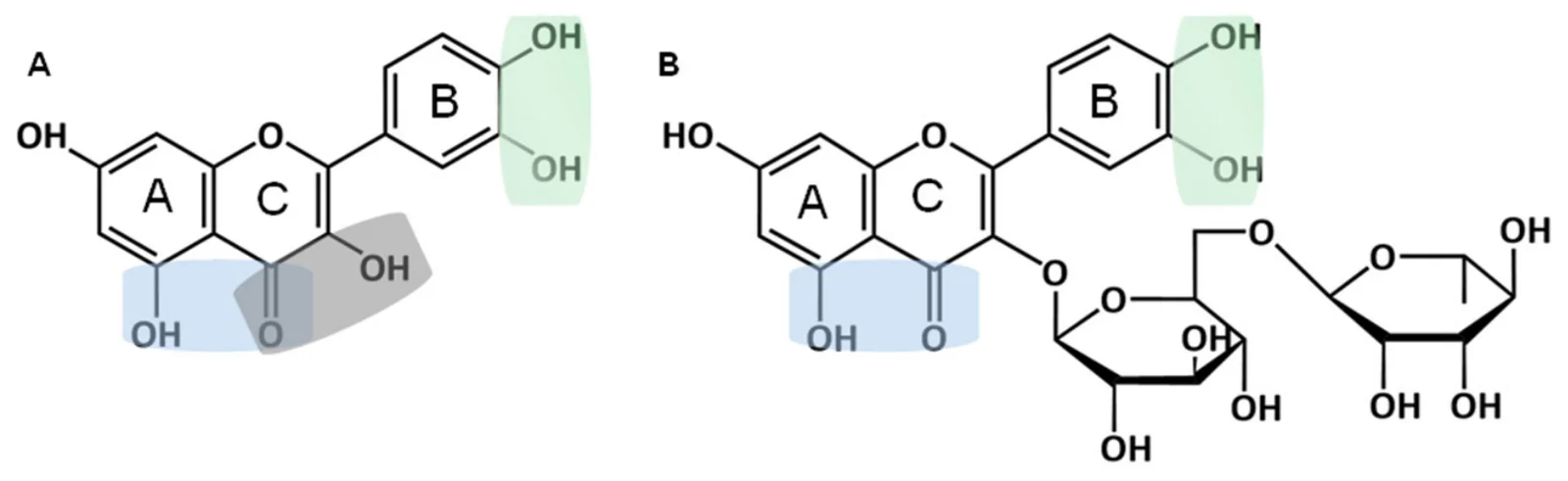
Chemical structures of quercetin (**A**) and rutin (**B**), with color highlights indicating typical coordination sites for metal ion binding.

**Figure 2:**
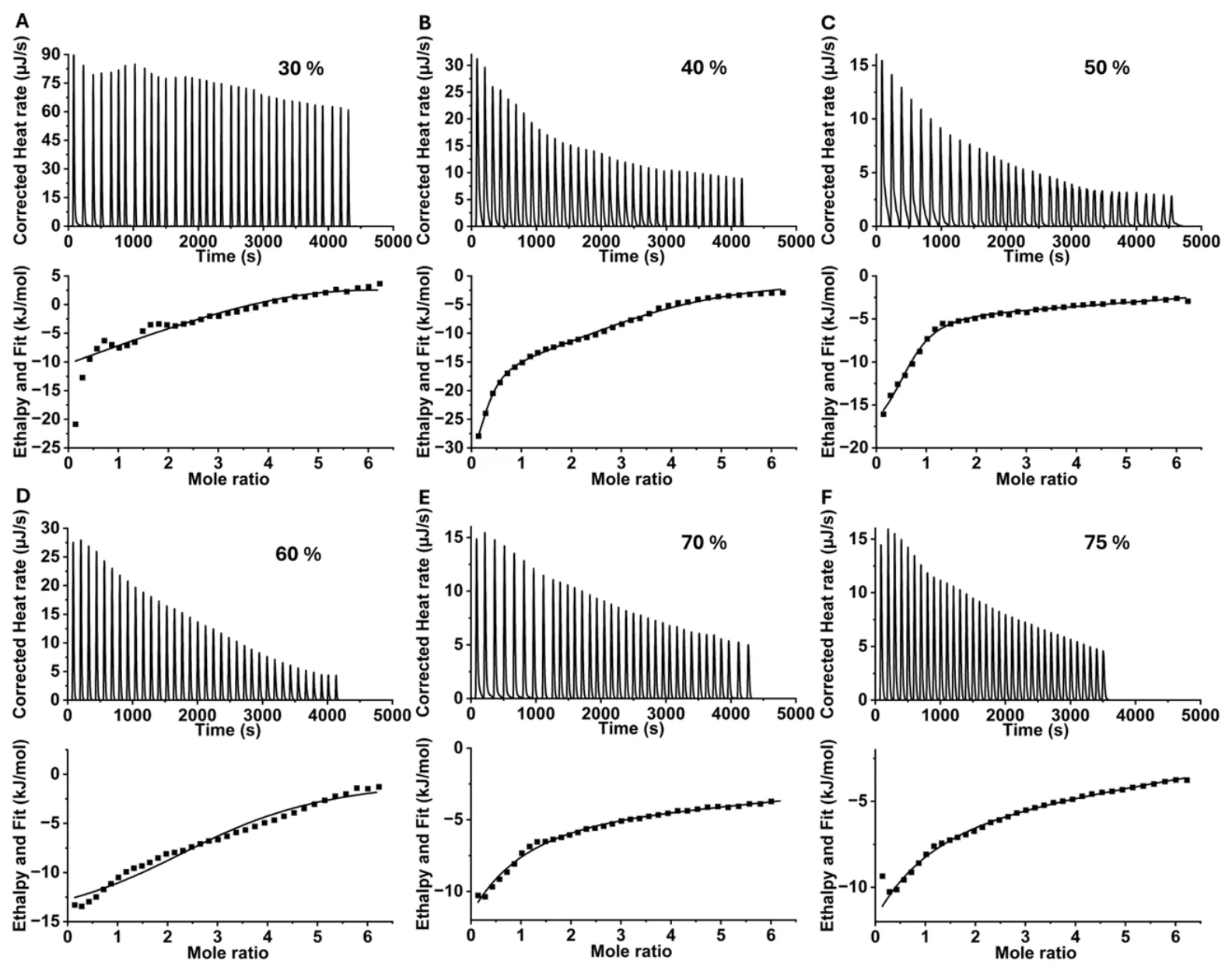
ITC thermograms showing the raw heat rate as a function of time (upper panels) and the corresponding integrated enthalpy change (Δ*H*) as a function of molar ratio, together with fitted binding curves (lower panels). Experiments were performed by sequentially injecting 2.5 μL of 15 mM Fe(III) aqueous solution into 1.5 mM quercetin prepared in ethanol–water mixtures containing (**A**) 30%, (**B**) 40%, (**C**) 50%, (**D**) 60%, (**E**) 70%, and (**F**) 75% ethanol (*v*/*v*). Each experiment was independently repeated three times, and Black squares indicate the experimental ITC data points, while the solid line represents the corresponding best-fit curve.

**Figure 3:**
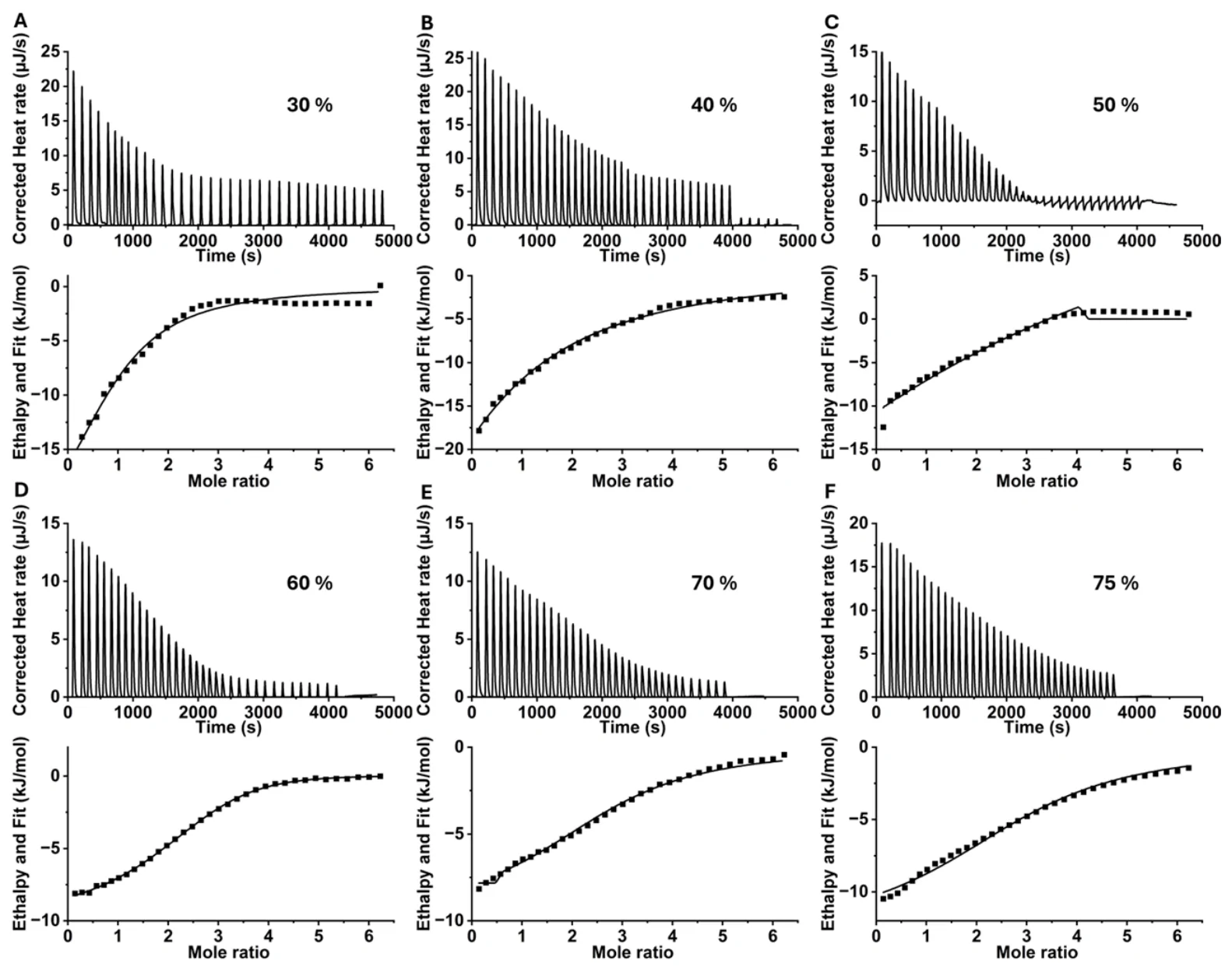
ITC thermograms showing the raw heat rate as a function of time (upper panels) and the corresponding integrated enthalpy change (Δ*H*) as a function of molar ratio, together with fitted binding curves (lower panels). Experiments were performed by sequentially injecting 2.5 μL of 15 mM Fe(III) aqueous solution into 1.5 mM rutin prepared in ethanol–water mixtures containing (**A**) 30%, (**B**) 40%, (**C**) 50%, (**D**) 60%, (**E**) 70%, and (**F**) 75% ethanol (*v*/*v*). Each experiment was independently repeated three times, and Black squares indicate the experimental ITC data points, while the solid line represents the corresponding best-fit curve.

**Figure 4:**
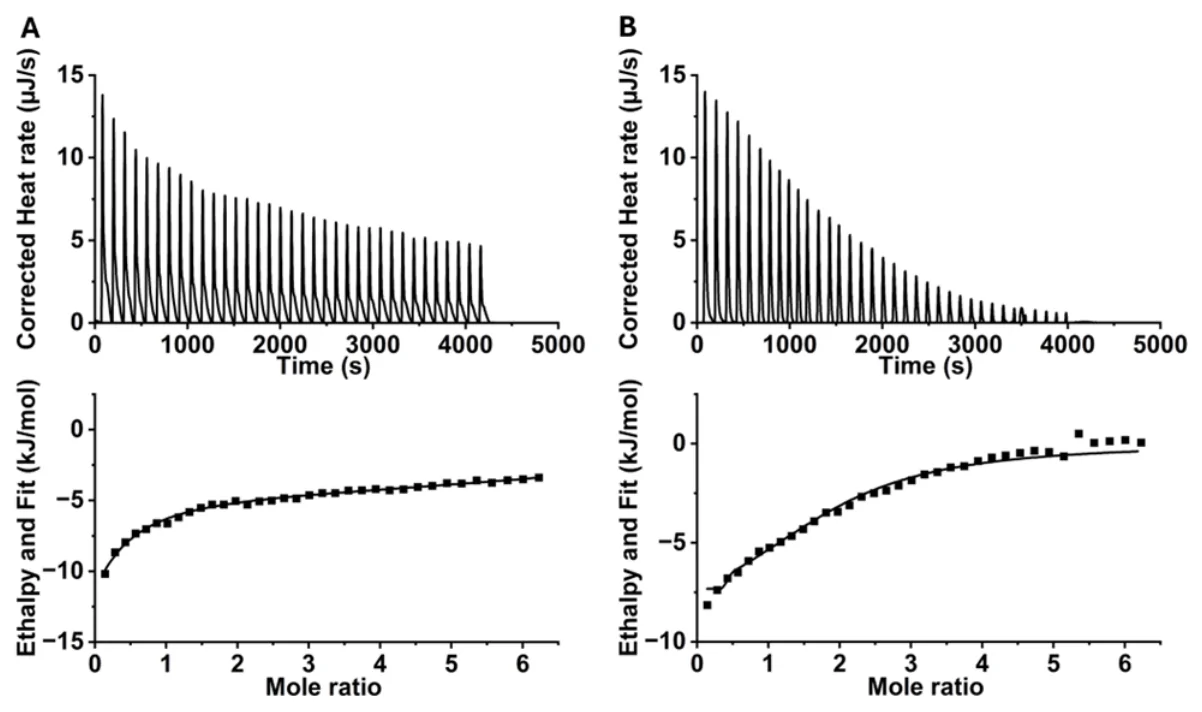
Representative ITC thermograms for quercetin (**A**) and rutin (**B**) at 37 °C using 60% ethanol solvent. The upper panels show raw heat rate versus time, and the lower panels display the integrated enthalpy as a function of the molar ratio. Here, Black squares indicate the experimental ITC data points, while the solid line represents the corresponding best-fit curve.

**Figure 5:**
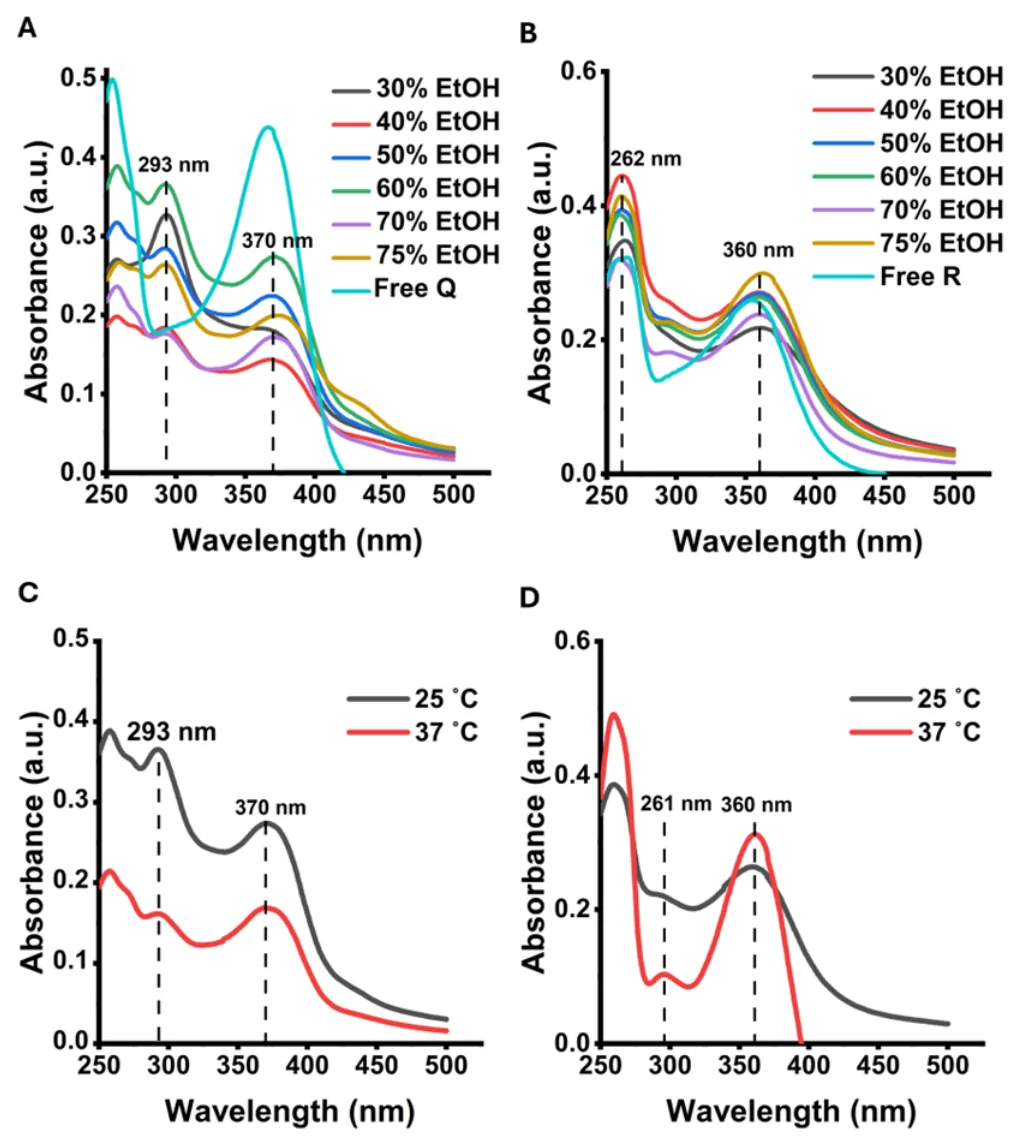
Normalized UV–vis absorbance spectra of the ITC reactions illustrating the effects of solvent composition and temperature on the interactions of quercetin and rutin with Fe(III). (**A**) Quercetin spectra obtained in ethanol–water mixtures containing 30–75% ethanol, with a characteristic absorption maximum near 293 nm and 370 nm. (**B**) Rutin spectra under the same solvent conditions, showing a distinct spectral feature near 360 nm and solvent-dependent changes in peak position, spectral intensity and shape. (**C**) Temperature-dependent spectra of quercetin–Fe(III) reactions measured at 25 and 37 °C, both exhibiting an absorption maximum near 293 nm and 370 nm. (**D**) Temperature-dependent spectra of rutin–Fe(III) reactions at 25 and 37 °C, showing pronounced differences in the spectral profile and absorbance near 261 nm and a shared peak at 360 nm.

**Table 1:** Apparent thermodynamic parameters derived from the interactions of quercetin and rutin with Fe(III) in a 60% ethanol–water mixture at 25 and 37 °C. Parameters were obtained by nonlinear fitting of the ITC binding isotherms using a multiple-site model with two fitted interaction sites. *K*_d_ is the apparent dissociation constant; Δ*H* is the apparent molar enthalpy change (kJ mol^−1^); and Δ*S* is the apparent molar entropy change (J/mol·K). Values are reported as parameter estimate ± fitting standard error. The reported uncertainties represent the standard errors from the nonlinear model fit and do not represent standard deviations from independent experimental replicates.

Flavonoid	Temperature (°C)	*K*_d1_ (M)	*K*_d2_ (M)	*n*_1_	*n*_2_	Δ*H*_1_ (kJ mol^−1^)	Δ*H*_2_ (kJ mol^−1^)	Δ*S*_1_ (J mol^−1^ K^−1^)	Δ*S*_2_ (J mol^−1^ K^−1^)
Quercetin	25	1.0 × 10^−3^ ± 1 × 10^−9^	(1.0 ± 0.2) × 10^−3^	0.1 ± 1	3 ± 1	100 ± 200	−20 ± 40	309	−2
	37	(1.0 ± 0.4) × 10^−3^	(8 ± 6) × 10^−5^	10 ± 2	0.1 ± 0.2	−5.0 ± 0.6	−60 ± 40	41	−125
Rutin	25	(2.1 ± 0.6) × 10^−4^	(1.0 ± 0.4) × 10^−3^	2.6 ± 0.9	1 ± 2	−10 ± 1	10 ± 100	36	80
	37	(1.0 ± 0.7) × 10^−3^	(2 ± 9) × 10^−8^	1.7 ± 1.0	0.4 ± 1.1	−10 ± 200	0 ± 300	27	122

## Data Availability

The original contributions presented in this study are included in this article. Further inquiries can be directed to the corresponding author.

## Supplementary Materials

The following supporting information can be downloaded at https://www.mdpi.com/article/10.3390/biom16101526/s1, Figure S1: Solubility of Quercetin in Ethanol–Water Mixtures; Figure S2: Solubility of Rutin in Ethanol–Water Mixtures; Figure S3: Solubility of Iron(III) Chloride in Ethanol–Water Mixtures; Figure S4: ITC Evaluation of Solvent-Related Heat Contributions: DMSO versus Ethanol; Figure S5: Reverse Titration of Quercetin into Fe(III): Assessment of ITC Experimental Configuration; Figure S6: ITC Dilution Thermograms of Rutin in Ethanol–Water Mixtures; Figure S7: ITC Dilution of Quercetin in Ethanol–Water Mixtures; Figure S8: ITC Hydrolysis Thermograms of Fe(III) in Ethanol-Water Mixtures; Figure S9: ITC Thermographs of Quercetin and Rutin dilution and Fe(III) hydrolysis in 37 °C; Table S1: Thermodynamics of Flavonoid–Fe(III) complexation in Other Solvent Percentage; Figure S10: Replicate ITC thermograms for flavonoid–Fe^3+^ complexation at 25 °C; Figure S11: Replicate ITC thermograms for flavonoid–Fe^3+^ complexation at 25 °C; Table S2: Apparent pH Values of Flavonoid and Fe(III) Reaction Solutions.

## Abbreviations

The following abbreviations are used in this manuscript:

ITC	Isothermal titration calorimetry
FTIR	Fourier transform infrared spectrophotometry
UV-Vis	Ultraviolet-visible
DMSO	Bimethyl sulfoxide
DI	Deionized
EtOH	Ethanol
NMR	Nuclear magnetic resonance
BSA	Brovine serum albumin
P-gp	P-glycoprotein
GLUT5	Glucose transporter member 5
