# Supplementary material for: Elucidating the Molecular Interactions of Flavonoid–Metal Complexes via Isothermal Titration Calorimetry
Source: Biomolecules. 2026 Oct 20;16(10):1526. doi: 10.3390/biom16101526 (PMC13702711; doi:10.3390/biom16101526)
Supplement: Supplementary file 1 [file biomolecules-16-01526-s001.zip › biomolecules-4586741-supplementary.pdf]

Elucidating the Molecular Interactions of Flavonoid–Metal Complex by Isothermal Titration Calorimetry

Martin Guerrero [1], Alex Kim [1], Colby Braden [1], and Yuping Bao [1,*]

[1] Department of Chemical and Biological Engineering, The University of Alabama, Tuscaloosa, AL 35487

* Correspondence: ybao@eng.ua.edu

1. Preparation of Quercetin and Rutin solution in Ethanol–Water Mixtures

The injecting solutions of quercetin and rutin (1.5 mM) were prepared by diluting 10 mM flavonoid stock solutions (10 mM) in 100% ethanol in an appropriate amount of water to obtain ethanol–water mixtures containing 30%, 40%, 50%, 60%, 70%, and 75% ethanol (v/v) and allowed to equilibrate overnight. The solutions were visually evaluated for clarity, turbidity, and evidence of phase separation. The quercetin solutions became progressively clearer with increasing ethanol concentration, with the 75% ethanol sample appearing the most transparent, indicating improved quercetin solubility (**Figure S1**). In contrast, decreasing the ethanol content increased turbidity, particularly in the 30–50% ethanol mixtures, consistent with reduced solubility and the onset of precipitation or suspended-particle formation. These observations demonstrate a strong dependence of quercetin solubility on solvent composition, with higher ethanol fractions favoring dissolution and more aqueous conditions promoting phase separation and particle formation.

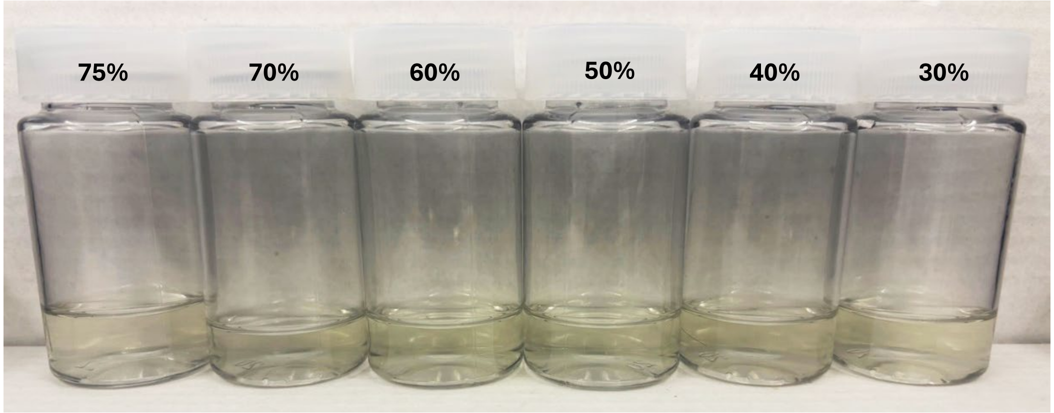

Figure S1. Visual assessment of quercetin solubility in ethanol–water mixtures. The solutions became progressively more turbid with decreasing ethanol content, indicating reduced quercetin solubility and increased formation of suspended particles under more aqueous conditions.

Figure S2 shows rutin solutions prepared in ethanol–water mixtures containing 75%, 70%, 60%, 50%, 40%, and 30% ethanol (v/v) after overnight equilibration. The samples containing 75–40% ethanol remain relatively clear, indicating that rutin is largely maintained in solution over this solvent composition range. In contrast, the 30% ethanol sample exhibits pronounced turbidity and a visible yellow dispersed/settled phase, consistent with substantially reduced rutin solubility and increased particle formation. These observations demonstrate a strong dependence of rutin solubility on solvent composition, with decreasing

ethanol content and increasing water fraction progressively reducing the solvent capacity for rutin and promoting phase separation or precipitation.

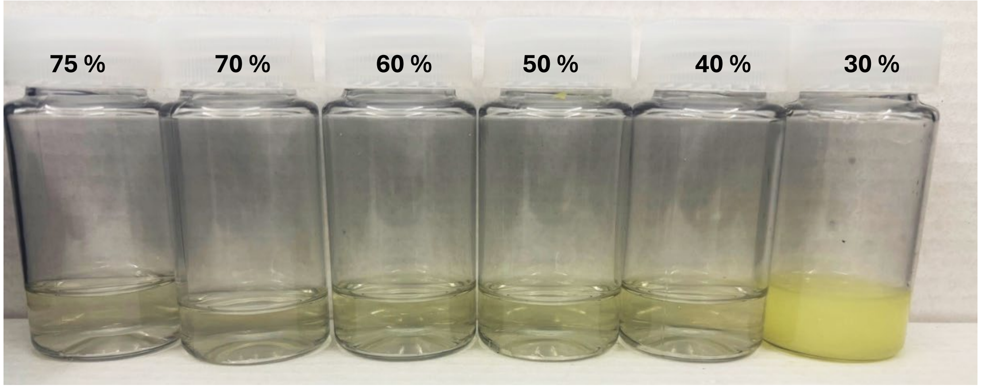

Figure S2. Representative photographs of rutin in ethanol–water mixtures containing 75%, 70%, 60%, 50%, 40%, and 30% ethanol (v/v). A visible yellow precipitate is observed in the 30% ethanol mixture, indicating reduced rutin solubility at higher water contents.

2. Preparation of Iron Chloride in Ethanol–Water Mixtures

Iron(III) chloride solutions were prepared in ethanol–water mixtures containing 75%, 70%, 60%, 50%, 40%, and 30% ethanol (v/v). As shown in Figure S3, all samples remained visually clear and homogeneous, with the characteristic orange-brown coloration of dissolved Fe(III) species. No obvious turbidity, suspended particles, or visible precipitation was observed across the investigated solvent compositions, suggesting that Fe(III) chloride remained well dispersed under these conditions. The intensity and appearance of the orange-brown coloration varied somewhat with ethanol concentration, consistent with changes in the solvent environment and potentially the coordination and hydrolysis equilibria of Fe(III) species. These visual observations indicate that, unlike the flavonoids, Fe(III) chloride did not exhibit pronounced solubility limitations over the range of ethanol–water compositions examined.

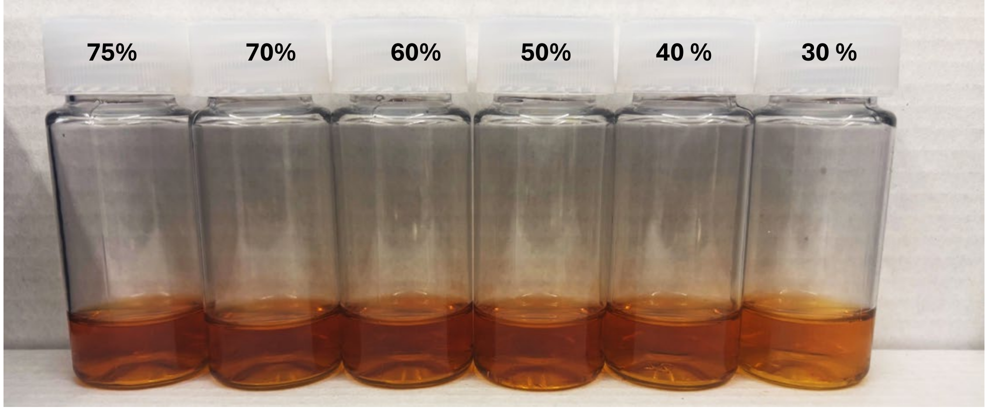

Figure S3. Representative photographs of iron(III) chloride in ethanol–water mixtures containing 75%, 70%, 60%, 50%, 40%, and 30% ethanol (v/v). All samples remained clear and homogeneous with no visible precipitation, indicating good solubility and solution stability over the investigated solvent composition range.

3. Selection of Solvent

The solvent is a critical factor for ITC experiments because of the contributions from desolvation and mixing heat during serial titrations. Ethanol and DMSO are the two most commonly used solvents for flavonoids; therefore, we first evaluated the mixing or dilution heat from these two solvents in the absence of reactants. In brief, solutions containing 5% (v/v) DMSO or ethanol (EtOH) in water were prepared and equilibrated overnight before analysis. Identical solvent solutions were loaded into the syringe and sample cell to minimize heat effects arising from differences in solvent composition. ITC measurements consisted of 35 consecutive injections (2.5 μL per injection) at a stirring speed of 125 rpm. As shown in **Figure S4A**, injections of the 5% DMSO solution produced relatively large heat-rate responses, with peak magnitudes that progressively decreased throughout the injection series. This pronounced and continuously changing response indicates substantial injection-associated heat effects in the DMSO-containing system, which could contribute to background signals during subsequent binding measurements. In contrast, the 5% EtOH solution produced substantially smaller heat-rate responses with relatively consistent peak profiles across the injection series (**Figure S4B**), indicating a lower and more stable background heat contribution. Based on these results, ethanol was selected as the preferred cosolvent for subsequent ITC experiments because it produced substantially lower and more stable background heat signals than DMSO, thereby minimizing interference from solvent-related thermal effects.

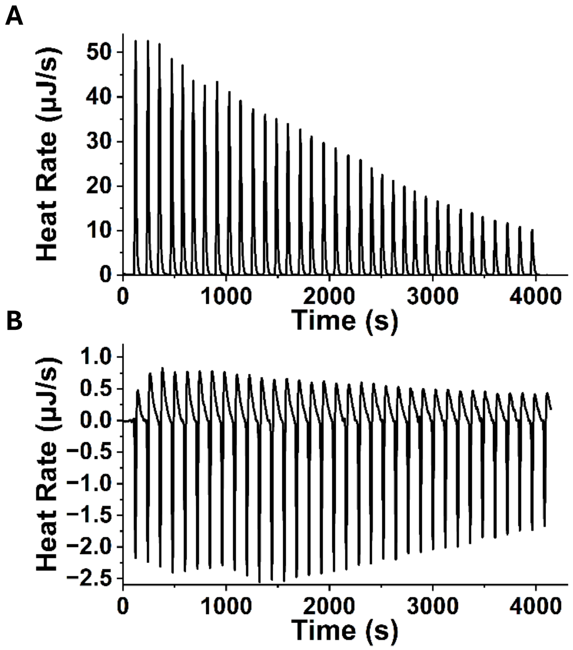

Figure S4. Comparison of solvent-associated heat effects during ITC measurements using 5% (v/v) DMSO (A) and 5% (v/v) ethanol (B) in water.

4. Evaluation of titration order

For Fe^{3+}-flavonoid interaction measurements, the titration can be performed either by injecting the flavonoid into an Fe(III)-containing sample cell or by reversing the configuration and injecting Fe(III) into the flavonoid-containing sample cell. To determine the more appropriate configuration for the present study, 1.5 mM quercetin in 50% (v/v) ethanol–water was titrated into 15 mM $FeCl_3$ prepared in the same

solvent. As shown in **Figure S5**, the reverse titration produced large and highly reproducible heat-rate peaks throughout the injection series. However, the peak magnitude increased during the initial injections and subsequently approached a nearly constant response, rather than progressively decreasing toward the baseline. Thus, the thermogram did not exhibit the characteristic concentration-dependent transition associated with progressive saturation of binding sites. In particular, there was no clearly defined sigmoidal transition from strong binding to a low, stable post-saturation heat signal that would support reliable determination of binding stoichiometry and thermodynamic parameters. Because of the lack of a well-defined saturation profile suitable for robust binding-model fitting, this titration configuration was not used for quantitative determination of the Fe^{3+}–quercetin thermodynamic parameters.

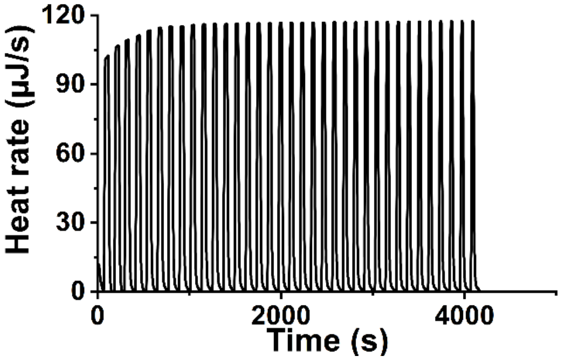

Figure S5. ITC thermogram obtained from titrating 1.5 mM quercetin in 50% (v/v) ethanol–water into 15 mM $FeCl_3$ in the same solvent.

5. Various control experiments

ITC measures the total heat associated with each injection, which may include contributions from solvent mixing, dilution, changes in solute solvation, and the desired molecular binding reaction. To characterize these non-binding heat contributions and establish appropriate background controls for the Fe(III)–flavonoid binding experiments, a series of control measurements was performed under different solvent conditions. To evaluate the effects of flavonoid solubility and solvent composition on the calorimetric response, 300 µL of 1.5 mM flavonoid solution was loaded into the sample cell and titrated with 250 µL of the corresponding ethanol–water solvent. Measurements were performed using ethanol concentrations ranging from 30% to 75% (v/v). The resulting thermograms demonstrated a clear dependence of the observed heat response on solvent composition and flavonoid solubility (**Figures S6 and S7**). At higher ethanol concentrations, the injection peaks were relatively consistent and returned reproducibly toward the baseline, indicating more homogeneous solutions and stable dilution behavior. In contrast, lower ethanol concentrations produced greater variability in peak profiles and baseline responses, consistent with increased contributions from non-binding processes. These effects were particularly evident at 30% ethanol, where visual inspection of the corresponding rutin sample showed pronounced turbidity and a visible yellow dispersed/settled phase (**Figure S2**), consistent with reduced rutin solubility. The associated irregular calorimetric responses may therefore reflect additional thermal contributions from processes such as dissolution, aggregation, or precipitation, making it difficult to distinguish these effects from the heat associated with molecular binding.

To account for the heat associated with $FeCl_3$ dilution and Fe(III) hydrolysis in the solvent system, control experiments were performed by titrating the 15 mM $FeCl_3$ solution into the corresponding blank solvent mixtures without flavonoid under otherwise identical experimental conditions **(Figure S8**). The resulting

$FeCl_3$ thermograms exhibited a strong dependence on ethanol concentration. Titration into higher ethanol concentrations, such as 60% and 70%, produced relatively consistent and regular endothermic injection profiles. In contrast, mid-range concentrations like 40% and 50% ethanol yielded complex, multiphasic calorimetric responses throughout the injection series, while 75% ethanol produced an entirely exothermic dilution profile.

To further characterize the baseline thermodynamic contributions of the individual experimental components, additional control experiments were performed by titrating iron chloride, quercetin, and rutin directly into the blank buffer system (**Figure S9**). As observed in panel A, the dilution of iron chloride produced significant, consistent endothermic peaks, reinforcing the necessity of accounting for Fe^{3+} hydrolysis and solvent interactions during background subtraction. In contrast, the dilution of quercetin into the buffer yielded a minimal, near-zero calorimetric response (**Figure S9B**), indicating that the background dilution heat for free quercetin is practically negligible under these conditions. Rutin, however, exhibited moderate, consistent endothermic dilution peaks (**Figure S9C**). By independently profiling the dilution heats of both the metal titrant and the individual flavonoids, the specific thermal contributions of solvent mixing, solute dilution, and background hydrolysis can be accurately distinguished from the heat of the desired molecular binding reaction.

Overall, these control experiments demonstrate that solvent composition and flavonoid solubility can substantially influence the measured ITC response. Higher ethanol concentrations provided more homogeneous solutions and more reproducible background signals, whereas lower ethanol concentrations increased the potential for solubility-related artifacts. These findings were therefore used to guide the selection of solvent conditions and appropriate background subtraction for subsequent Fe(III)-flavonoid binding measurements.

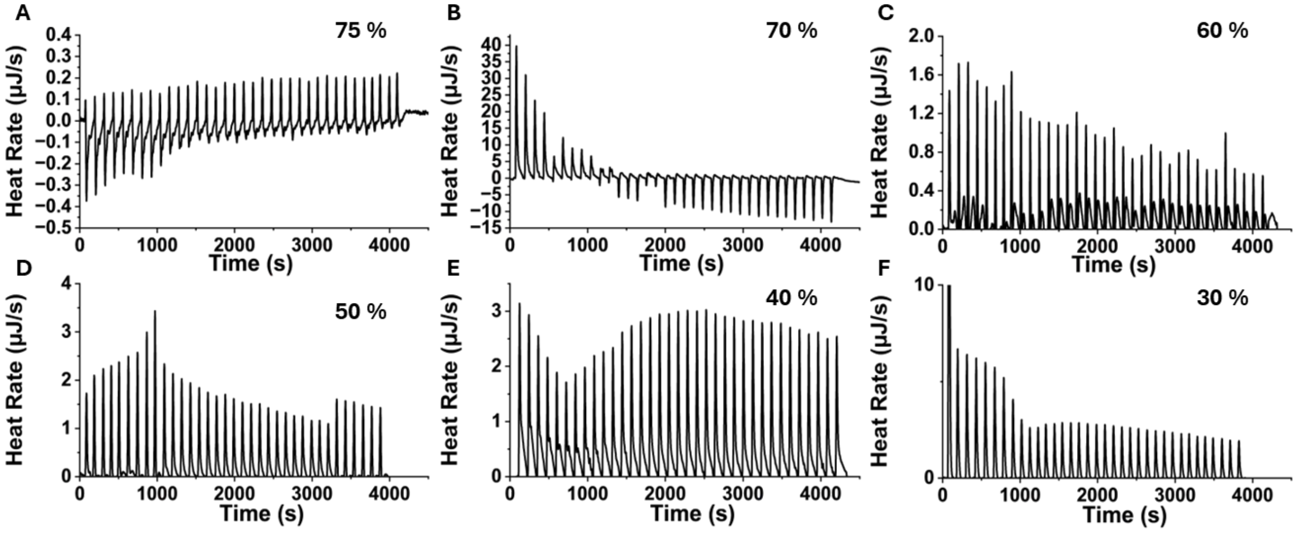

Figure S6: Control ITC thermograms showing dilution heats of rutin in ethanol. Rutin (panels A-F) was titrated into buffer at various ethanol (EtOH) concentrations to assess solvent-dependent dilution effects.

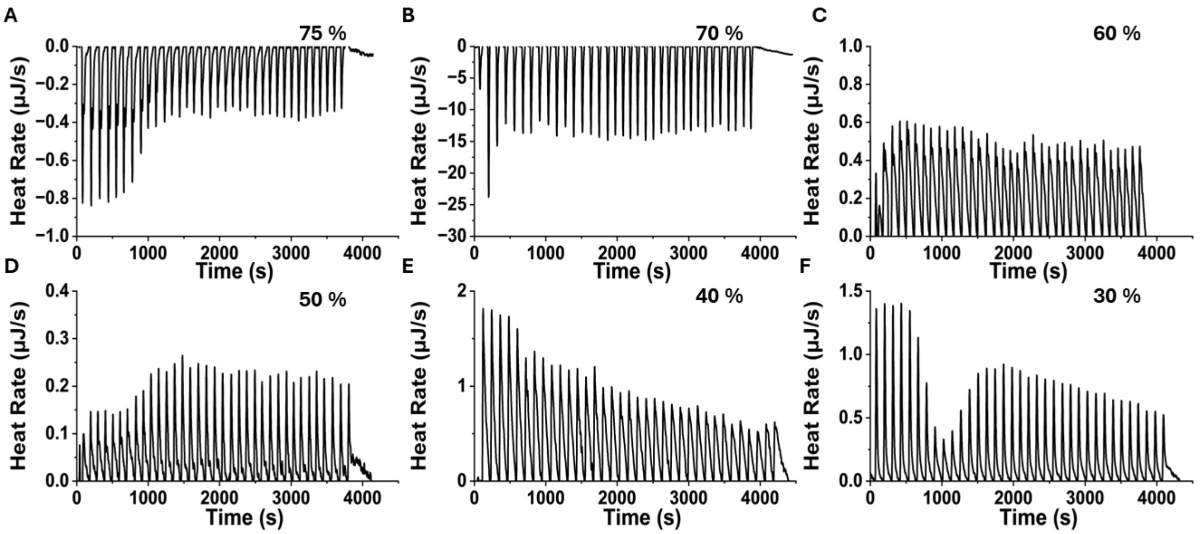

Figure S7. Control ITC thermograms showing dilution heats of quercetin in ethanol. Quercetin (panels A-F) was titrated into buffer at various ethanol (EtOH) concentrations to assess solvent-dependent dilution effects.

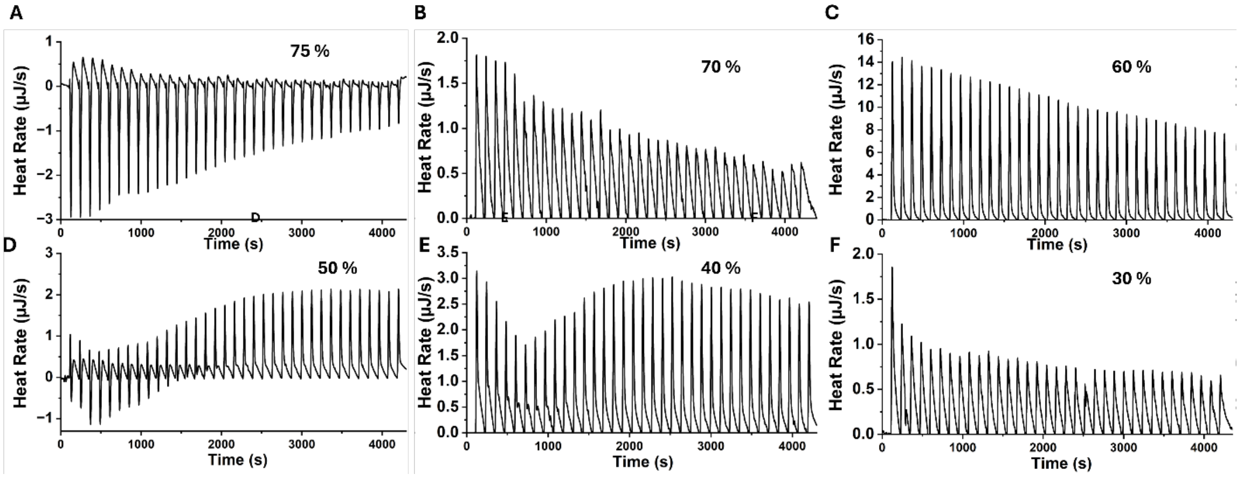

Figure S8. Control ITC thermograms showing dilution heats of iron chloride in ethanol. Iron chloride (panels A-F) was titrated into buffer at various ethanol (EtOH) concentrations to assess solvent-dependent dilution effects.

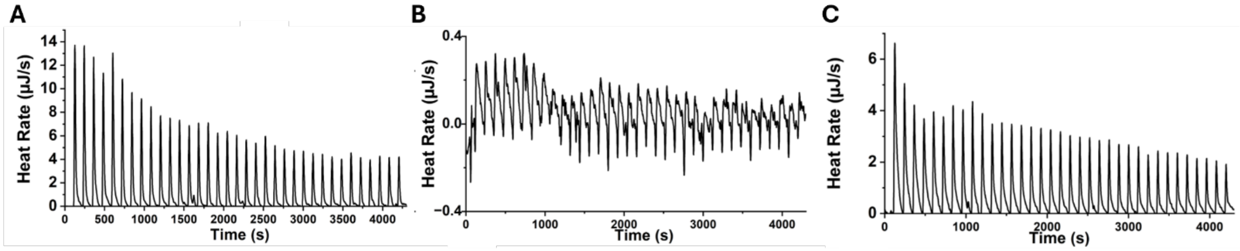

Figure S9. Control ITC thermograms showing dilution heats of iron chloride, quercetin, and rutin in ethanol. Iron chloride (panel A), quercetin (panel B), and rutin (panel C) were titrated into buffer at various ethanol (EtOH) concentrations to assess solvent-dependent dilution effects.

6. Thermodynamics of Flavonoid-Fe (III) complexation in Other Solvent Percentage

Isothermal titration calorimetry (ITC) enables the direct thermodynamic quantification of molecular interactions by extracting the binding affinity, stoichiometry, enthalpy, and entropy from the integrated heat responses. Following the subtraction of the non-binding heat contributions established in the control experiments, the background-corrected isotherms for the Fe(III)–flavonoid interactions were fitted to a two-site binding model. To evaluate the thermodynamic driving forces of these complexation events across different solvent environments, the specific dissociation constants (K_d), reaction stoichiometries (n), enthalpy changes (ΔH), and entropy changes (ΔS) were determined for both quercetin and rutin.

Table S1. ITC thermodynamic parameters for the complexation of quercetin and rutin with Fe^{3+}. Measurements were performed across various ethanol (EtOH) concentrations, and data were fitted using a multiple-binding-site model.

Flavonoid	Solvent (% EtOH)	K_{d1} (M)	K_{d2} (M)	n_1	n_2	ΔH_1 (kJ/mol)	ΔH_2 (kJ/mol)	ΔS_1 (J/mol·K)	ΔS_2 (J/mol·K)
Quercetin	30	$4.1 \times 10^{-4} \pm 2 \times 10^{-9}$	$4.7 \times 10^{-4} \pm 4 \times 10^{-9}$	3 ± 1	4 ± 2	-200 ± 100	160 ± 30	-552	610
	40	$(1 \pm 8) \times 10^{-3}$	$(4 \pm 5) \times 10^{-5}$	3.4 ± 0.3	0.3 ± 0.1	-18 ± 2	-40 ± 40	-4	-47
	50	$(1.0 \pm 0.3) \times 10^{-3}$	$(2 \pm 1) \times 10^{-5}$	8 ± 1	0.70 ± 0.08	-4.6 ± 0.8	-17 ± 3	42	34
	60	$1.0 \times 10^{-3} \pm 1 \times 10^{-9}$	$(1.0 \pm 0.2) \times 10^{-3}$	0 ± 1	3 ± 1	100 ± 200	-20 ± 40	309	-2
	70	$5.5 \times 10^{-5} \pm 3 \times 10^{-9}$	$3.1 \times 10^{-4} \pm 1 \times 10^{-8}$	0.1 ± 0.3	10 ± 2	-110 ± 70	-3.7 ± 0.5	-274	55
	75	$(2.0 \pm 0.4) \times 10^{-4}$	$(1.0 \pm 0.1) \times 10^{-3}$	0.1 ± 0.3	10 ± 1	-200 ± 100	-5 ± 1	-463	42
Rutin	30	$(0.1 \pm 2) \times 10^{-2}$	$1.0 \times 10^{-3} \pm 2 \times 10^{-9}$	1.0 ± 0.6	0.1 ± 0.6	-50 ± 200	200 ± 100	-109	728
	40	$(2 \pm 2) \times 10^{-8}$	$(5 \pm 4) \times 10^{-9}$	5.9 ± 0.7	0.5 ± 0.3	0.5 ± 0.8	-90 ± 30	152	-150
	50	$(0.2 \pm 1) \times 10^{-8}$	$(0.3 \pm 1) \times 10^{-8}$	0.9 ± 0.6	3.1 ± 0.7	-80 ± 40	19 ± 9	-105	227
	60	$(2.1 \pm 0.6) \times 10^{-4}$	$(1.0 \pm 0.4) \times 10^{-3}$	2.6 ± 0.9	1 ± 2	-10 ± 1	10 ± 100	36	80
	70	$(1 \pm 2) \times 10^{-9}$	$(0.1 \pm 4) \times 10^{-2}$	0.5 ± 0.3	2.5 ± 0.2	-7.8 ± 0.4	-9.2 ± 0.7	146	26
	75	$(0.1 \pm 2) \times 10^{-2}$	$(1 \pm 1) \times 10^{-3}$	2 ± 2	1 ± 2	-10 ± 30	-20 ± 60	30	-16

7. Reproducibility and Temperature Dependence of ITC Measurements

To validate the reliability of the calculated thermodynamic parameters, independent replicate ITC titrations were performed for the complexation of both quercetin and rutin with Fe(III). To further evaluate the thermal stability and physiological temperature of these binding interactions, the replicate measurements were conducted at both standard ambient temperature (25 °C) and physiological temperature (37 °C). The resulting thermograms and corresponding two-site binding model fits exhibit consistent heat signatures, binding affinities, and stoichiometric ratios across multiple independent trials. This reproducibility confirms that the observed thermodynamic profiles represent stable, consistent molecular interactions rather than stochastic experimental artifacts.

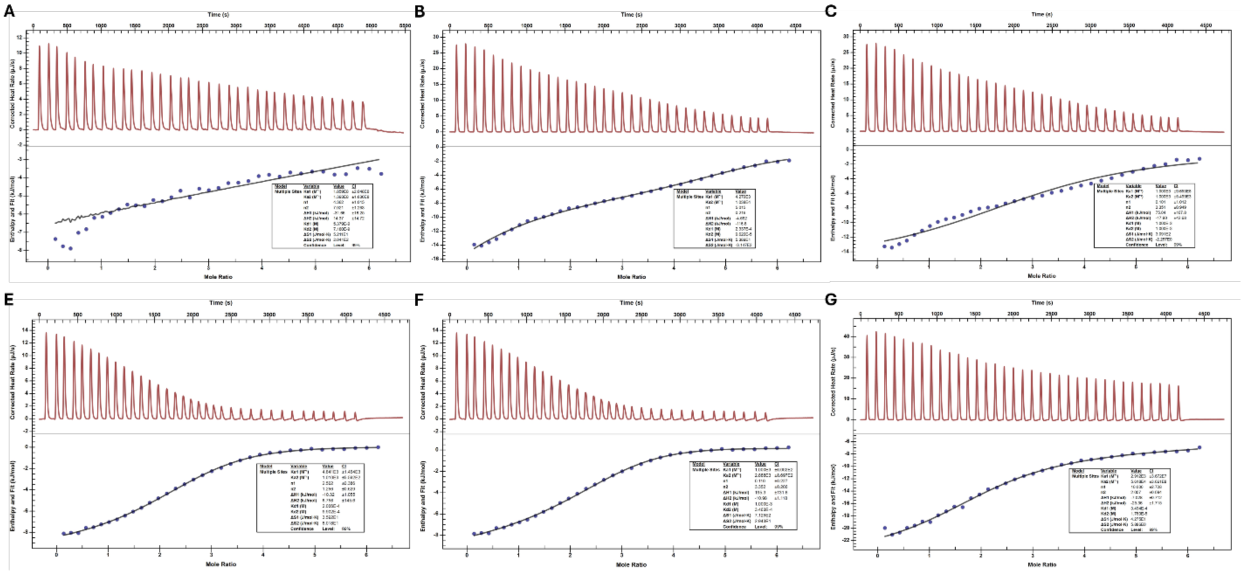

Figure S10. Replicate ITC thermograms for flavonoid–Fe^{3+} complexation at 25 °C. The raw heat rate peaks (top) and integrated enthalpy fits (bottom) are shown for multiple independent trials of quercetin (Panels A-C) and rutin (Panels E-G) in 60% ethanol. The consistency across replicates demonstrates the reproducibility of the extracted thermodynamic parameters.

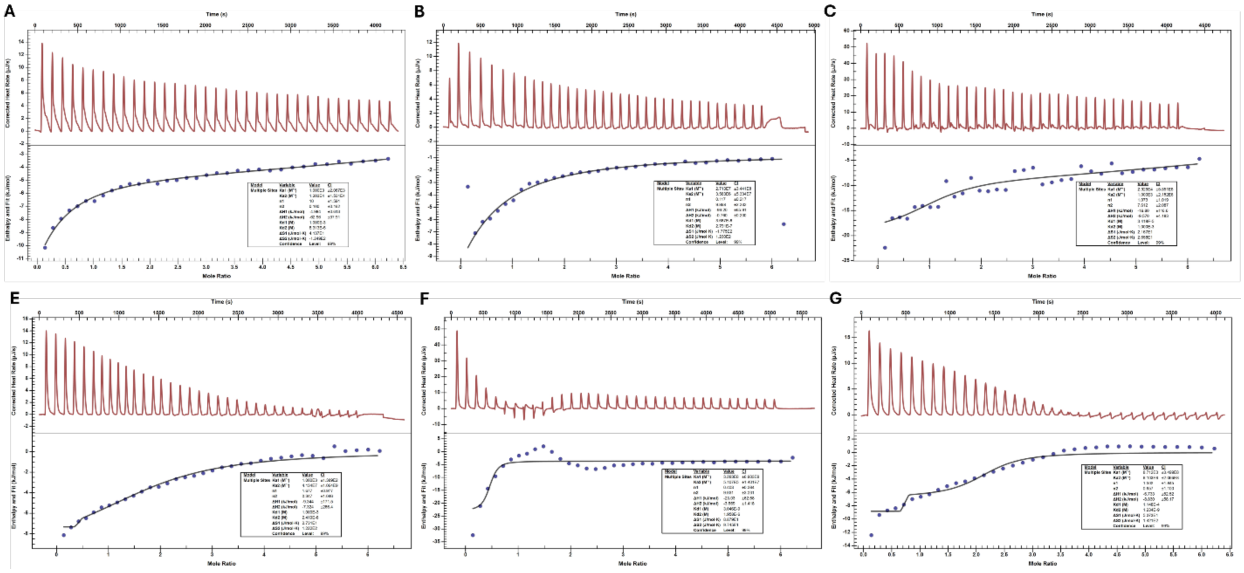

Figure S11: Replicate ITC thermograms for flavonoid–Fe^{3+} complexation at 37 °C. The raw heat rate peaks (top) and integrated enthalpy fits (bottom) are shown for multiple independent trials of quercetin (Panels A-C) and rutin (Panels E-G) in 60% ethanol. The consistency across replicates demonstrates the reproducibility of the extracted thermodynamic parameters.

8. The pH change before and after ITC

To account for the influence of protonation events and pH alterations during complexation, the calorimetric parameters obtained from the ITC measurements are explicitly defined as apparent thermodynamic parameters, rather than quantities representing an isolated molecular binding reaction. To monitor these solution changes, the pH of the individual flavonoid and $FeCl_3$ solutions was measured prior to titration, and the pH of representative reaction mixtures was recorded following the addition of Fe^{3+} under standard ITC conditions. The resulting measurements are

summarized in Table S2. Importantly, because these experiments were conducted in binary ethanol–water mixtures, all reported measurements are designated as apparent pH values corresponding to their specific solvent environments.

Table S2. Apparent pH values of individual flavonoid and $FeCl_3$ solutions prior to titration, and of representative reaction mixtures following the addition of Fe (III) under standard ITC experimental conditions. Because measurements were conducted in binary ethanol–water mixtures, all reported values are designated as apparent pH values corresponding to their specific solvent environments.

Compound	Solvent (% EtOH)	pH before titration	pH after titration
**Quercetin**	**30**	**4.2**	**2.5**
	**40**	**4.1**	**2.4**
	**50**	**4.1**	**2.4**
	**60**	**4.3**	**2.4**
	**70**	**4.4**	**2.4**
	**75**	**4.1**	**2.4**
**Rutin**	**30**	**4.4**	**2.6**
	**40**	**4.8**	**2.5**
	**50**	**4.8**	**2.5**
	**60**	**4.6**	**2.5**
	**70**	**4.4**	**2.4**
	**75**	**4.5**	**2.4**
**$FeCl_3$**	**30**	**2.1**	
	**40**	**2.0**	
	**50**	**2.0**	
	**60**	**2.0**	
	**70**	**1.9**	
	**75**	**1.9**	
